# Comparative microbiome analysis in cystic fibrosis and non-cystic fibrosis bronchiectasis.

**DOI:** 10.1186/s12931-024-02835-w

**Published:** 2024-05-18

**Authors:** Heryk Motta, Júlia Catarina Vieira Reuwsaat, Fernanda Cortez Lopes, Graciele Viezzer, Fabiana Caroline Zempulski Volpato, Afonso Luís Barth, Paulo de Tarso Roth Dalcin, Charley Christian Staats, Marilene Henning Vainstein, Lívia Kmetzsch

**Affiliations:** 1 Laboratório de Biologia Molecular de Patógenos, Centro de Biotecnologia, Universidade Federal do Rio Grande do Sul, Porto Alegre, Brazil; 2 Departamento de Biofísica, Instituto de Biociências, Universidade Federal do Rio Grande do Sul, Porto Alegre, Brazil; 3 Programa de Pós-Graduação em Biologia Celular e Molecular, Universidade Federal do Rio Grande do Sul, Porto Alegre, Brazil; 4 Serviço de Pneumologia, Hospital de Clínicas de Porto Alegre, Porto Alegre, Brazil; 5 Laboratório de Pesquisa em Resistência Bacteriana, Centro de Pesquisa Experimental, Hospital de Clínicas de Porto Alegre, Porto Alegre, Brazil; 6 Departamento de Medicina Interna, Faculdade de Medicina, Universidade Federal do Rio Grande do Sul, Porto Alegre, Brazil; 7 Laboratório de Microrganismos de Importância Médica e Biotecnológica, Centro de Biotecnologia, Universidade Federal do Rio Grande do Sul, Porto Alegre, Brazil

**Keywords:** Bronchiectasis, Cystic fibrosis, Non-cystic fibrosis, Microbiome, Resistome

## Abstract

**Background:**

Bronchiectasis is a condition characterized by abnormal and irreversible bronchial dilation resulting from lung tissue damage and can be categorized into two main groups: cystic fibrosis (CF) and non-CF bronchiectasis (NCFB). Both diseases are marked by recurrent infections, inflammatory exacerbations, and lung damage. Given that infections are the primary drivers of disease progression, characterization of the respiratory microbiome can shed light on compositional alterations and susceptibility to antimicrobial drugs in these cases compared to healthy individuals.

**Methods:**

To assess the microbiota in the two studied diseases, 35 subjects were recruited, comprising 10 NCFB and 13 CF patients and 12 healthy individuals. Nasopharyngeal swabs and induced sputum were collected, and total DNA was extracted. The DNA was then sequenced by the shotgun method and evaluated using the SqueezeMeta pipeline and R.

**Results:**

We observed reduced species diversity in both disease cohorts, along with distinct microbial compositions and profiles of antimicrobial resistance genes, compared to healthy individuals. The nasopharynx exhibited a consistent microbiota composition across all cohorts. Enrichment of members of the *Burkholderiaceae* family and an increased Firmicutes/Bacteroidetes ratio in the CF cohort emerged as key distinguishing factors compared to NCFB group. *Staphylococcus aureus* and *Prevotella shahii* also presented differential abundance in the CF and NCFB cohorts, respectively, in the lower respiratory tract. Considering antimicrobial resistance, a high number of genes related to antibiotic efflux were detected in both disease groups, which correlated with the patient’s clinical data.

**Conclusions:**

Bronchiectasis is associated with reduced microbial diversity and a shift in microbial and resistome composition compared to healthy subjects. Despite some similarities, CF and NCFB present significant differences in microbiome composition and antimicrobial resistance profiles, suggesting the need for customized management strategies for each disease.

**Supplementary Information:**

The online version contains supplementary material available at 10.1186/s12931-024-02835-w.

## Background

Bronchiectasis is a chronic pulmonary condition characterized by the irreversible dilation of the bronchi and recurrent pulmonary infections, frequently accompanied by persistent cough and sputum production [1]. This chronic respiratory condition can be classified into two main categories: cystic fibrosis (CF) bronchiectasis and non-cystic fibrosis bronchiectasis (NCFB) [2]. Bronchiectasis can arise from various causes, including congenital conditions such as CF and primary ciliary dyskinesia, as well as acquired conditions like post-infective bronchiectasis, immune deficiency-associated bronchiectasis, chronic obstructive pulmonary disease (COPD)-related bronchiectasis, and idiopathic etiology [3].

There has been an upward trend in the number of bronchiectasis cases since 2004, which has led to a substantial increase in hospitalizations [4, 5], while CF is estimated to affect at least 100,000 people worldwide [6]. At the end of 2020, approximately 6,112 individuals were registered in the Brazilian Cystic Fibrosis Registry. Among these, 10.4% are in Rio Grande do Sul state, where this research was conducted [7].

Bronchiectasis pathobiology was first described as a vicious cycle of tissue damage comprising infection, inflammation triggered by the infection, lung damage, mucus stasis, and subsequent reinfection [8]. An updated concept considers this cycle a vicious vortex, wherein all the factors interact to promote disease progression. The pathophysiology of CF bronchiectasis results from mutations in the cystic fibrosis transmembrane conductance regulator (*CFTR*) gene, which is responsible for chloride and water transport, leading to the production of thick mucus. The challenge in clearing this more viscous mucus creates a favorable environment for infections, perpetuating the vicious vortex [9].

Given that infection plays a pivotal role in both CF and NCFB, the investigation of microbiome composition in these conditions holds significant value in shedding light on disease progression. The healthy lung microbiome is characterized by a dynamic, diversified, and low-density microbiota. It is primarily shaped by three critical factors: the influx of microbes from the upper respiratory tract, the efflux of organisms via coughing or the host’s immune response, and microbial replication within the lungs [10]. In CF bronchiectasis, a reduction in taxonomic diversity and an increased prevalence of pathogenic taxa are strongly associated with disease severity markers and an unfavorable prognosis [11]. Regarding NCFB, studies are contradictory regarding the correlation between reduced taxa and disease severity [12, 13]. These divergent findings emphasize the potential advantages of a microbiome-directed therapeutic approach that dynamically modulates the lung microbiome in a taxon-dependent manner. This approach demonstrates superiority over empirical antibiotic therapy, as antibiotic efficacy is contingent upon the presence of specific taxa, having a limited effect in their absence [14].

Despite advances in studies of lung microbiome composition and its correlations with CF and NCFB prognosis, most such studies rely on 16S rRNA partial gene sequencing, thereby limiting the investigations to bacterial composition [15]. Metagenomic next-generation sequencing (mNGS), also known as the shotgun approach, has the notable advantage of enabling total DNA sequencing and thereby, the acquisition of comprehensive information on bacterial, fungal, archaeal, and viral composition, while also allowing for resistome analysis [16]. Despite its advantages, its significant limitations in a clinical setting, such as the high time demand, complexity of analysis, bioinformatic technical requirements, and sequencing costs, warrant consideration [17]. Despite these difficulties, commercial tests utilizing these technologies are emerging, with promising results [18]. Studies simultaneously investigating both CF and NCFB are limited [19] and, given the role of the upper respiratory tract in shaping the lung microbiome [10], it is essential to collect and analyze sputum and nasopharyngeal samples concurrently.

This study aimed to analyze the differences in the lung and nasopharyngeal microbiome compositions among three groups: CF and NCFB patients and healthy subjects. Additionally, resistome profiles were investigated, focusing on individuals from the disease groups with a history of chronic antibiotic usage. This research seeks to enhance our understanding of the respiratory microbiome’s role in CF and NCFB and offers insights into potential therapeutic strategies and improved management approaches for these conditions.

## Methods

### Patient recruitment criteria

Individuals with CF and NCFB were recruited from the outpatient CF clinic at Hospital de Clínicas de Porto Alegre (HCPA), Brazil. All enrolled patients were in stable clinical condition at the time of sampling and had not experienced any respiratory infections in the preceding 4 weeks. For CF patients, inclusion criteria consisted of a chloride level ≥ 60 mmol/L as per the sweat test and two confirmed pathological mutations in the *CFTR* gene. Conversely, NCFB patients were included based on chloride levels < 30 mmol/L as per the sweat test, an absence of pathological *CFTR* gene mutations, and a clinical diagnosis of NCFB by medical staff. Healthy subjects were recruited from among HCPA staff and the Universidade Federal do Rio Grande do Sul (UFRGS) community, with the requirement of having no comorbidities. All participants fell within the age range of 18 to 60 years, and recruitment and sample collection occurred between December 2021 and October 2022. Sample size determination was guided by relevant studies in the field with a similar design [20–23].

### Specimen collection and processing

Nasopharyngeal samples were obtained from each participant’s nostril using a flexible, sterile, soft-tipped swab according to Centers for Disease Control and Prevention (CDC) guidelines [24]. The collected swabs were then placed in conical tubes containing 3 mL of sterile saline for preservation. Sputum induction was performed as per the guidelines of the Brazilian Ministry of Health, involving a 20-minute administration of 3% hypertonic saline through ultrasonic nebulizers. Patients were subsequently prompted to cough up sputum into plastic containers. For unviable samples, an additional 10-minute nebulization with hypertonic saline was administered, followed by another attempt at sputum expectoration. Induced sputum samples and swabs were stored at −80 °C until DNA extraction. DNA was extracted from sputum with the ZymoBIOMICS DNA Miniprep Kit (Zymo Research, Irvine, CA, USA) following the manufacturer’s instructions. Nasopharyngeal swab samples were thawed, homogenized for 1 minute in a vortex, and transferred to tubes containing 200 µL of glass beads. Processing was done in the FastPrep-24™ 5G bead beater (MP Biomedicals, Santa Ana, CA, USA), following the same specifications as for sputum samples. Subsequent extraction was performed using 25:24:1 phenol:chloroform:isoamyl alcohol (Invitrogen, Carlsbad, CA, USA), followed by DNA precipitation using ethanol (Merck, Darmstadt, Germany), according to a previously published protocol [25]. The DNA was then resuspended in 50 µL UltraPure™ DNase/RNase-Free Distilled Water (Invitrogen, Carlsbad, CA, USA). For the negative control, a background sample was processed; DNA extraction was performed as above, but no quantifiable DNA was obtained, rendering it unsuitable for sequencing.

### DNA Sequencing

Sequencing was performed at the Laboratório Central de Tecnologias de Alto Desempenho (LaCTAD), an affiliate of the Universidade Estadual de Campinas (UNICAMP) located in São Paulo, Brazil. The process entailed generating paired-end reads, each consisting of 150 base pairs (2 × 150 bp), using the Illumina HiSeq 2500 instrument (Illumina, San Diego, CA, USA).

### Data processing

The FastQ raw files were subjected to cleaning and preprocessing using the fastp tool [26]. To eliminate human sequences, alignment against the human reference genome hg37dec_v0.1 was carried out using bowtie2 [27]. Unaligned sequences to the human genome were removed using SAMtools [28]. The SqueezeMeta pipeline was employed for tasks including contig assembly, functional annotation, taxonomic classification, and binning contigs into metagenome-assembled genomes [29]. Additionally, the Resistance Gene Identifier (RGI) tool was applied to detect resistance genes in the samples [30]. Data exploration and statistical analysis were conducted in R using the following packages: SQMtools [31] for importing SqueezeMeta data, phyloseq [32] for data manipulation and microbiome analysis, vegan [33] and agricolae [34] for ecological and statistical analyses, pairwiseAdonis [35] for pairwise comparisons, and DESeq2 [36] for differential abundance assessment. Adjusted* p* values lower than 0.01 were considered significant. Finally, ggplot2 [37] was employed for graphics creation and visualization.

### Statistical analysis

Statistical differences were analyzed using either ordinary one-way analysis of variance (ANOVA) followed by Tukey’s multiple comparison test or the Kruskal–Wallis test followed by Dunn’s post hoc test, depending on whether the data followed a normal distribution or not. Beta-diversity was assessed using Bray–Curtis principal coordinates analysis (PCoA) coupled with permutation multivariate analysis of variance (PERMANOVA). Differential abundance analysis was performed through pairwise comparison between the studied cohorts. Data were normalized using DESeq2, and the Wald test was applied.

## Results

### Cohort composition and sequencing data

A total of 35 subjects were enrolled, as outlined in Table 1. The NCFB cohort included 10 individuals, eight of whom had available matched sputum and nasopharyngeal samples. The CF cohort comprised 13 members, nine of whom had paired samples of sputum and nasopharyngeal swabs. None of the included CF patients were undergoing highly effective CFTR modulator therapy at the time of recruitment. In the healthy cohort, nine subjects had paired sputum and swab samples. Overall, of the 61 processed samples, 52 were paired, originating from 26 patients, while nine samples were unpaired. All samples were utilized for cohort comparisons regarding the upper or lower respiratory tract microbiome. However, for correlation assessments between the upper and lower respiratory tracts, only the 52 paired samples were used. Statistical analysis did not reveal any significant age differences among the cohorts (*p* value = 0.12). In total, 19 subjects were using antimicrobial agents continuously, with seven of them belonging to the NCFB cohort and 12 to the CF cohort (Table 1).

**Table 1:** Composition of the cohorts

	Cohort category			Healthy
	All subjects	NCFB	CF	
Number of subjects	35	10	13	12
Sex (female:male)	20:15	7:3	6:7	7:5
Mean age (SD)	29.6 (8.5)	31.7 (11.8)	26.5 (6.8)	31.3 (5.2)
Age range	18-59	22-59	18-31	23-39
Subjects continuously using antimicrobial agents	19	7	12	0

*NCFB* Non-CF bronchiectasis, *CF* Cystic fibrosis

Clinical data extracted from the patients’ records are provided in Table 2. Most patients in both disease cohorts had chronic *Pseudomonas aeruginosa* colonization. The antibiotic regimen included oral azithromycin and inhaled colistimethate, gentamicin, and tobramycin, representing a macrolide, a polymyxin, and two aminoglycosides, respectively, in addition to the oral antifungal agent itraconazole. Three individuals (be_05, cf_07, and cf_10) exhibited antimicrobial resistance profiles, as assessed using classical microbiological techniques (Table 2).

**Table 2:** Clinically relevant data of the NCFB (coded as *be*) and CF (coded as *cf*) cohorts

**Subject**	**Age**	**Sex**	**FVC**	**FEV1**	**FEV1/FVC**	**Chronic colonization**	**Long-term antimicrobial therapy drugs**	**Resistance identified through disk diffusion**
be_01	22	M	1.55	0.8	0.516	*-*	Azithromycin	-
be_02	24	M	-	-	-	*-*	-	-
be_03	23	F	2.13	1.44	0.676	*P. aeruginosa*	Polymyxin E	-
be_04	29	F	2.34	1.82	0.777	*P. aeruginosa*	Azithromycin	-
be_05	27	M	3.05	1.72	0.563	*P. aeruginosa, S. aureus*	Colistimethate	Cefepime, ceftazidime, ciprofloxacin, and piperacillin/tazobactam
be_06	25	F	3.13	2.43	0.776	*P. aeruginosa, S. aureus*	Azithromycin, gentamicin	-
be_07	32	F	1.77	1.26	0.711	*P. aeruginosa, Achromobacter* sp.	Colistimethate, azithromycin	-
be_08	59	F	1.1	0.72	0.654	*P. aeruginosa*	Azithromycin, polymyxin E	-
be_09	26	F	-	-	-	*-*	-	-
be_10	50	F	2.13	1.75	0.821	*-*	-	-
cf_01	31	F	2	1.45	0.725	*P. aeruginosa, S. aureus*	Azithromycin, polymyxin E, tobramycin, itraconazole	-
cf_02	27	F	2.64	2.07	0.784	*P. aeruginosa, S. aureus*	Azithromycin	-
cf_03	37	F	3.31	2.44	0.737	*P. aeruginosa, S. aureus*	Polymyxin E	-
cf_04	24	F	2.86	1.63	0.569	*-*	Azithromycin	-
cf_05	22	M	2.98	1.9	0.637	*P. aeruginosa, S. aureus*	Tobramycin	-
cf_06	24	M	3.01	1.77	0.588	*B. cepacia, S. aureus, P. aeruginosa*	Polymyxin E, azithromycin, itraconazole	-
cf_07	22	M	5.28	3.28	0.621	*S. aureus*	Itraconazole	Ciprofloxacin
cf_08	18	M	3.83	2.28	0.595	*B. cepacia, P. aeruginosa*	Azithromycin, tobramycin	-
cf_09	37	F	2.73	1.89	0.692	*B. cepacia*	Azithromycin	-
cf_10	37	F	2.56	2.34	0.914	*P. aeruginosa*	Polymyxin E	Cefepime
cf_11	19	M	4.11	3.09	0.751	*-*	Azithromycin, tobramycin	-
cf_12	27	M	4.6	3.38	0.734	*H. influenzae*	-	-
cf_13	19	M	4.12	3.06	0.742	*P. aeruginosa, S. aureus*	Azithromycin, tobramycin, polymyxin E	-

FVC, forced vital capacity (L); FEV1, forced expiratory volume in the first second (L); FEV1/FVC, FEV1 and FVC ratio; M, male; F, female; -, unavailable data

Overall, total DNA from 31 sputum and 30 nasopharyngeal samples was subjected to high-throughput sequencing. Following trimming, approximately 0.73% of the reads were eliminated. Human reads constituted nearly 99% of the total reads. Despite the high number of human reads, the sequencing coverage was sufficient to reach the plateau on the rarefaction curves, demonstrating adequate capture of the microbial community and resistance marker diversity in the samples (Figure S1). The data related to each sample can be found in supplementary Table_S1.

### Cystic fibrosis and non-cystic fibrosis bronchiectasis influence microbiota diversity in sputum

To understand the impact of CF and NCFB on microbiota, we conducted taxonomic profiling of the samples. The major microbial component of the lungs and nasopharynx was bacteria, representing 48% of cleaned reads. Viruses accounted for approximately 0.35% of cleaned reads, fungi for 0.05%, and archaea for 0.0001%. Detailed information is presented in supplementary Table_S2, Table_S3, and Table_S4.

**Fig. 1:**
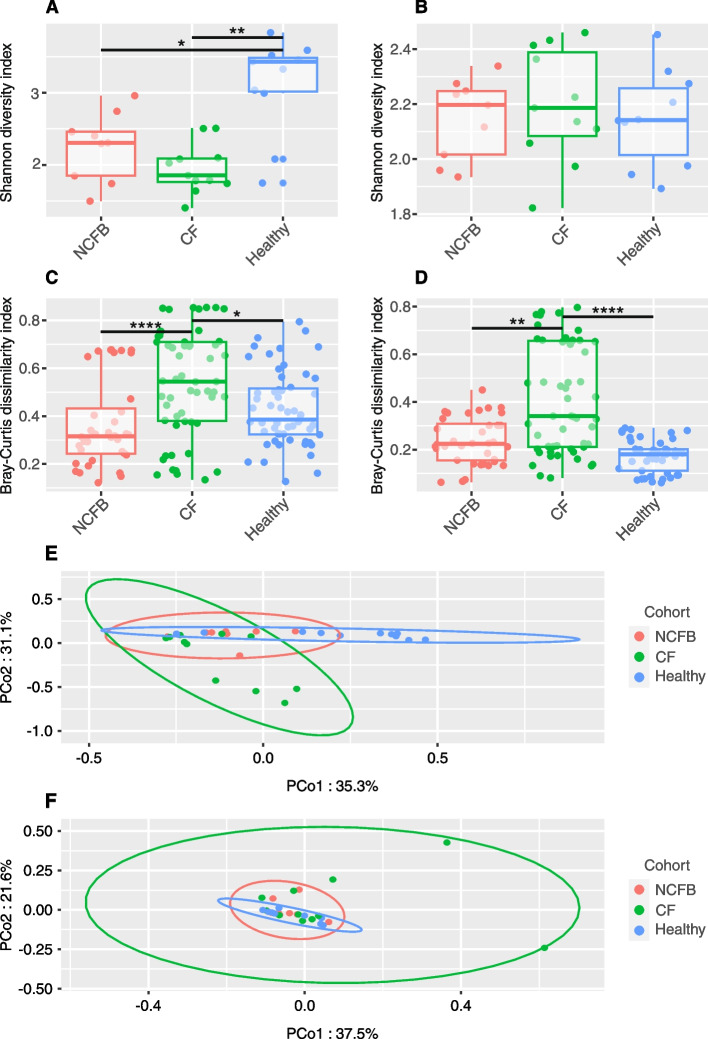
Diversity analysis in NCFB and CF patients and healthy subjects. **A** Alpha diversity in sputum microbiota. Sputum barplot illustrating Shannon diversity as a measure of alpha diversity. Healthy subjects display higher alpha diversity compared to individuals with CF and NCFB. **B** Alpha diversity in nasopharyngeal swab microbiota. Nasopharyngeal swab barplot illustrating Shannon diversity as a measure of alpha-diversity. No significant differences were observed among the three cohorts. **C** Interpersonal variation in sputum microbiota. Sputum barplot representing intra-cohort Bray–Curtis dissimilarity. The points denote pairwise Bray–Curtis distance combinations, revealing increased interpersonal variation within the CF cohort. **D** Interpersonal variation in nasopharyngeal swab microbiota. Nasopharyngeal swab barplot representing intra-cohort Bray–Curtis dissimilarity. The points denote pairwise Bray–Curtis distance combinations, revealing increased interpersonal variation within the CF cohort. **E** Sputum microbiota cluster analysis. PCoA of Bray–Curtis distances in sputum microbiota, showing the distinct separation of healthy subjects from disease cohorts. While the CF cohort exhibits less clustering than the NCFB group, there is a noticeable overlap between the two disease cohorts. **F** Nasopharyngeal swab microbiota cluster analysis. PCoA of Bray–Curtis distances in nasopharyngeal swab microbiota, showing clustered distribution among all three cohorts. Ellipses denote 95% confidence. Statistical significance was assessed using the Kruskal–Wallis test followed by Dunn’s post hoc test for all performed analyses as none assumed a normal distribution (* *P* ≤ 0.05, ** *P* ≤ 0.01, and **** *P* ≤ 0.0001)

To assess the influence of CF and NCFB on microbial composition, a diversity evaluation was performed. A comparison of Shannon’s index among the three cohorts revealed a diminished alpha diversity in both pathological cohorts compared to the healthy one in sputum samples, which represent the lower airways (Fig. 1A). However, this difference was not observed in nasopharyngeal samples, which represent the upper airways (Fig. 1B). The same pattern was observed when analyzing other alpha diversity indices such as Simpson and evenness (Figure S2).

To investigate the heterogeneity within cohorts, we compared subjects within the same cohort using the Bray–Curtis dissimilarity index. The CF cohort exhibited a significantly higher mean Bray–Curtis index compared to both NCFB and healthy cohorts (Fig. 1C and D), indicating a more substantial dissimilarity among individuals affected by CF. This pattern was observed in both sputum and nasopharyngeal swab samples.

PCoA, coupled with PERMANOVA comparisons (beta diversity), was applied to evaluate potential differences in microbiome community composition among CF, NCFB, and healthy subjects. Sputum samples exhibited noticeable stratification between the healthy and pathological cohorts, as shown in Fig. 1E. Healthy and NCFB subjects displayed a clustered distribution. In contrast, the CF cohort exhibited a lack of homogeneity, indicated by higher dispersion in the PCoA. Statistical differences were assessed using PERMANOVA, revealing that both CF and NCFB groups significantly differed from the healthy cohort (adjusted *p* values of 0.0003 and 0.0087, respectively). There was no significant difference between the CF and NCFB cohorts (adjusted *p* value = 0.1167). In contrast, the PCoA of nasopharyngeal samples indicated dispersed distribution regardless of the cohort, as shown in Fig. 1F. Individuals with CF also displayed a heterogeneous distribution, indicating a higher degree of dispersion. Subsequent PERMANOVA analysis confirmed that the nasopharyngeal composition of all three cohorts was not substantially different.

### Microbiota composition and diversity in sputum samples: contrasting impact of cystic fibrosis and non-cystic fibrosis bronchiectasis

**Fig. 2:**
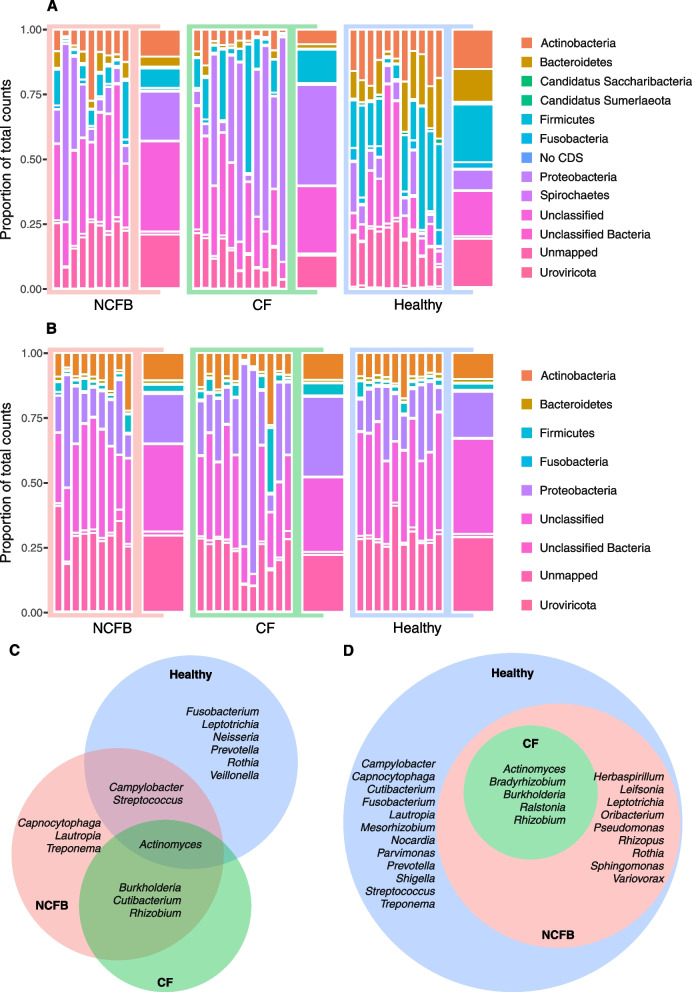
Phylum-level composition and core microbiota. **A** Sputum microbiota: Phylum-level composition of sputum microbiota in NCFB and healthy cohorts. Thinner bars represent individual subjects, while thicker bars represent the cohort average. **B** Nasopharyngeal swab microbiota: Phylum-level composition of nasopharyngeal swab microbiota as shown in (**A**). **C** and **D** Core microbiota at the genus level in the sputum (**C**) and nasopharyngeal swab (**D**) samples shown as Euler diagrams depicting unique and shared components

To elucidate disparities in diversity across the three cohorts, a comprehensive compositional analysis was conducted. This analysis involved the computation of the relative abundance of each sample and cohort, followed by the generation of phylum-level plots for both sputum and nasopharyngeal specimens. The lower respiratory tract samples exhibited more pronounced distinctions (Fig. 2A), while the nasopharyngeal samples demonstrated a higher degree of compositional consistency across all three cohorts, as illustrated in Fig. 2B.

**Fig. 3:**
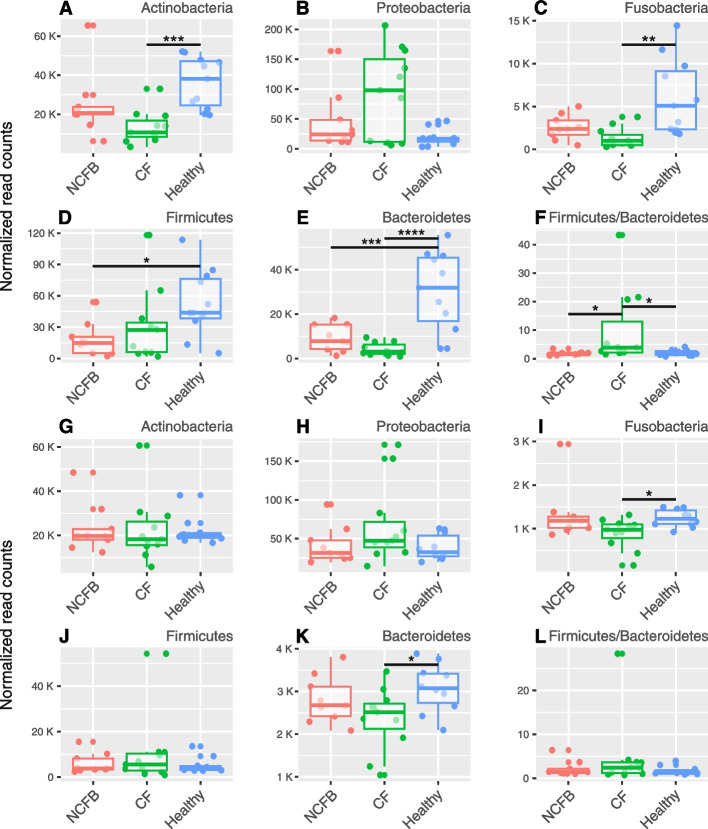
Phylum composition comparison. **A**-**F** Sputum analysis: Comparison of phylum-level composition between NCFB, CF, and healthy cohorts. **G**-**L** Nasopharyngeal swab analysis: Comparison of phylum-level composition in nasopharyngeal swabs for the above cohorts. Statistical significance: The Kruskal–Wallis test was applied followed by Dunn’s post hoc test, except for Bacteroidetes analysis, which used ordinary one-way ANOVA followed by Tukey’s multiple comparison test (* *P* ≤ 0.05, ** *P* ≤ 0.01, *** *P* ≤ 0.001, and **** *P* ≤ 0.0001)

To achieve a more precise compositional evaluation of the microbiome among the cohorts, the core components of the microbiota at the genus level were determined, considering a prevalence of 99.9% and an abundance of at least 0.1% (Fig. 2C and D). The phylum-level distinctions were assessed using normalized read counts for each phylum as the basis for comparison (Fig. 3). We selected prevalent phyla for our comparative analysis, including Actinobacteria, Proteobacteria, Fusobacteria, Firmicutes, and Bacteroidetes, as well as the Firmicutes to Bacteroidetes (F/B) ratio.

Sputum samples exhibited substantial dissimilarities in both the core composition of the microbiota (Fig. 2C) and phylum-level comparisons (Fig. 3A-F). In contrast, the core microbiota components of nasopharyngeal samples in both diseases were included in the healthy core microbiota (Fig. 2D), and phylum-level differences were observed in only a limited subset (Fig. 3G-L).

In sputum samples, the healthy cohort exhibited a core composition comprising the *Actinomyces, Campylobacter, Fusobacterium, Leptotrichia, Neisseria, Prevotella, Rothia, Streptococcus,* and *Veillonella* genera, associated with higher counts of Actinobacteria (Fig. 3A), Fusobacteria (Fig. 3C), Firmicutes (Fig. 3D), and Bacteroidetes (Fig. 3E). The CF cohort exhibited only four genera in its core microbiota: *Actinomyces*, also present in healthy subjects, and *Burkholderia*, *Cutibacterium*, and *Rhizobium*, which were exclusive to the disease cohort. The NCFB cohort displayed the *Actinomyces*, *Burkholderia*, *Campylobacter*, *Cutibacterium*, *Rhizobium*, *Streptococcus*, *Capnocytophaga*, *Lautropia*, and *Treponema* genera, with the last three being exclusive to the NCFB core. The F/B ratio was significantly higher in the CF cohort compared to both NCFB and healthy cohorts. The healthy and NCFB cohorts exhibited a near 1:1 F/B ratio, indicating a proportional composition (Fig. 3F). This distinction in the F/B ratio was the sole dissimilarity observed between the CF and NCFB cohorts.

The comparison of nasopharyngeal phyla and core microbiota revealed fewer disparities among the cohorts. Fusobacteria and Bacteroidetes were both enriched in the healthy cohort in comparison to the CF cohort (Fig. 3I and K). Additionally, the NCFB cohort did not show any major discernible differences from the CF and healthy cohorts.

The correlation of phyla with patients’ lung function was assessed through linear regression of forced expiratory volume in 1 second (FEV1)—an established measure of lung function—with phylum counts. None of the models demonstrated a correlation with lung function (Figure S3 and S4).

### Species differential abundance across cohorts

Differential species abundance analysis was conducted through pairwise comparisons among the three cohorts. To enhance the precision of the analysis, only species exhibiting a log_2_FoldChange (log_2_FC) above 2 were considered. The volcano plots illustrating each comparison can be found in Figure S5, and the significant differential abundance data of each sample can be accessed in supplementary Table_S5, Table_S6, and Table_S7.

**Fig. 4:**
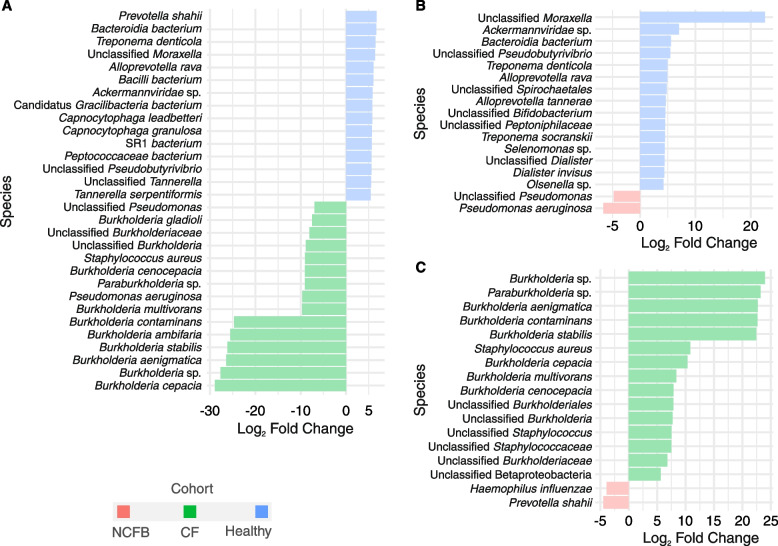
Pairwise differential abundance analysis in sputum samples. **A** Healthy *vs.* CF: Differential abundance analysis comparing healthy subjects to individuals with CF. **B** Healthy *vs.* NCFB: Differential abundance analysis comparing healthy subjects to individuals with NCFB. **C** CF *vs.* NCFB: Differential abundance analysis comparing the CF and NCFB cohorts. DESeq2 was employed for the analysis, with significance defined by an adjusted *p* value below 0.01. The figures represent the top 15 differentially abundant species, with a log_2_FC of 2 for all the comparisons

When the sputum samples of the healthy cohort were juxtaposed with those of the CF cohort (Fig. 4A), the species enriched in the healthy cohort predominantly comprised commonly found components of the oral microbiome, such as members of genus *Prevotella*. Enrichment of *Ackermannviridae* sp. phage was also observed in the healthy cohort. The CF cohort exhibited an array of enriched species, including known pathogens from the genera *Pseudomonas*, *Staphylococcus*, and *Xanthomonas* (associated with *Stenotrophomonas*), and numerous entries from the order Burkholderiales.

Comparing the healthy and NCFB cohorts revealed a lower number of microorganisms exhibiting differential abundance relative to the comparison of the CF and healthy cohorts (Fig. 4B). The enriched species identified in healthy subjects included those from the *Alloprevotella*, *Moraxella*, *Treponema*, *Dialister*, *Leptotrichia*, *Mogibacterium*, and *Olsenella* genera. Conversely, NCFB subjects exhibited the enrichment of only two microorganisms: an unclassified *Pseudomonas* and *P. aeruginosa*.

When the CF and NCFB cohorts were compared (Fig. 4C), the CF cohort exhibited enrichment of bacteria belonging to the order Burkholderiales, indicating the primary distinction between them. Additionally, *Staphylococcus aureus* was significantly enriched in the CF group. In contrast, NCFB subjects displayed a differential abundance of *Haemophilus influenzae* and *Prevotella shahii* compared to those with CF. In the nasopharyngeal differential abundance comparison conducted among the three cohorts, only *Corynebacterium propinquum* displayed significant enrichment in the NCFB group in relation to the healthy cohort.

### Differential functional analysis based on metagenomics data

**Fig. 5:**
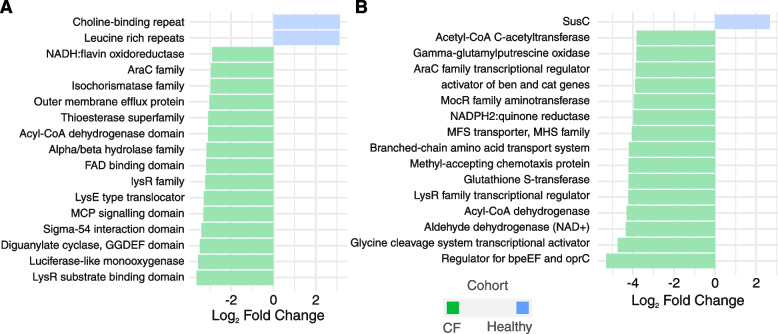
Pairwise functional differential abundance analysis comparing healthy and CF cohorts. **A** and **B**. Differential abundance analysis comparing functional enriched processes in the microbiome in Pfam (**A**) and KO (**B**). Green bars represent the CF cohort and blue bars represent the healthy cohort. DESeq2 was employed for the analysis, with significance defined by an adjusted* p* value below 0.05. The figures represent the top 15 significantly differentially abundant processes

To explore how specific microbial functions may contribute to the disease’s manifestations and outcomes, we conducted a differential functional analysis based on the microbiome profiles. Results from the Kyoto Encyclopedia of Genes and Genomes (KEGG) Ontology (KO), Clusters of Orthologous Groups (COG), and Pfam annotations were compared among cohorts. There were differences in the identified function abundance, specifically between the CF and healthy cohorts, within the KO and Pfam annotations. Three accessions appeared enriched in the healthy cohort (choline-binding repeat, leucine-rich repeat, and SusC), while a series of processes related to basic metabolism and virulence were enriched in the CF cohort (Fig. 5). A more detailed display of the functional analysis data can be found in supplementary Table_S8 and Table_S9.

### Antimicrobial resistance genes differ across cohorts in the lower respiratory tract

**Fig. 6:**
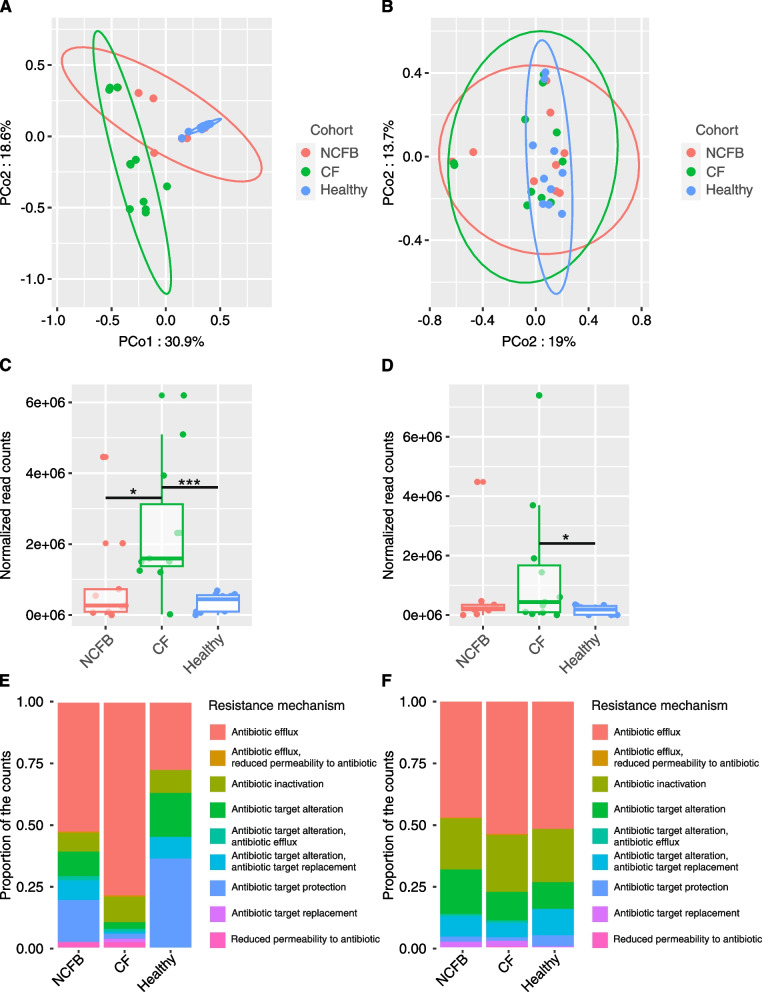
Resistome analysis. **A** Sputum resistome cluster analysis: PCoA of Bray–Curtis distances in the sputum resistome, showing distinct separation of NCFB, CF, and healthy cohorts. **B** Nasopharyngeal swab resistome cluster analysis: PCoA of Bray–Curtis distances in the nasopharyngeal swab resistome, revealing no significant differences between cohorts. **C** Sputum antimicrobial resistance gene incidence: The CF cohort exhibits a higher incidence of resistance genes compared to the NCFB and healthy cohorts. **D** Nasopharyngeal swab antimicrobial resistance gene incidence: The CF cohort has a higher incidence of resistance genes than the healthy cohort. **E** Sputum antimicrobial resistance mechanism composition: NCFB and CF cohorts display increased antibiotic efflux, while the healthy cohort exhibits a homogeneous composition. **F** Nasopharyngeal swab antimicrobial resistance mechanism composition: NCFB, CF, and healthy cohorts show a similar distribution of resistance mechanisms. Ellipses denote 95% confidence. Statistical significance was assessed by the Kruskal–Wallis test followed by Dunn’s post hoc test (* *P* ≤ 0.05 and *** *P* ≤ 0.001)

Considering the impact of antimicrobial resistance on treatment failure and the spread of resistant strains, our investigation included resistome profile determination, encompassing all cohorts herein analyzed. The composition of identified antimicrobial resistance genes is presented in supplementary Table_S10 and Table_S11. The lower respiratory tract samples displayed distinct compositional variations among the three cohorts under study, a pattern that was clearly discernible through the PCoA visualization (Fig. 6A). This observed disparity was confirmed through PERMANOVA analysis. The adjusted *p* values for the comparisons were 0.0003 for the healthy *vs.* CF cohort, 0.0069 for the healthy *vs.* NCFB cohort, and 0.0402 for the CF *vs.* NCFB cohort. Among the nasopharyngeal samples, no significant differences were observed (Fig. 6B).

Considering the lower respiratory tract, a significantly higher frequency of resistance genes within the CF cohort than in both the NCFB and healthy cohorts was found (Fig. 6C). This difference was also identified in nasopharyngeal samples, in which the CF cohort exhibited a significantly higher frequency of resistance genes compared to the healthy cohort. However, when compared to the NCFB cohort, no statistical significance was observed (Fig. 6D).

Distinct patterns of resistance mechanisms were discernible among the three cohorts, as depicted in Fig. 6E and F. In the lower respiratory tract, enrichment of antibiotic efflux mechanisms was evident in both disease cohorts, characterized by an uneven distribution of other mechanisms. In contrast, healthy subjects exhibited a more uniform composition of resistance mechanisms within this context (Fig. 6E). The dynamics of resistance mechanisms in nasopharyngeal samples differed from those observed in sputum samples, with a similar distribution among the three cohorts (Fig. 6F).

The vast majority of identified resistance genes in the disease cohorts were found in *P. aeruginosa*, as indicated in Tables S10 and S11. The variations observed in resistome analysis can be solely attributed to pathogen composition. In the case of *P. aeruginosa*, PERMANOVA analysis did not reveal a significant difference between disease cohorts. The top 10 most abundant resistance genes identified per cohort are displayed in Table 3. In the CF cohort, multidrug resistance was observed for *Burkholderia cepacia* and *Burkholderia pseudomallei*, while in the NCFB cohort, the resistance genes were associated with *Haemophilus influenzae*. It is noteworthy that *Burkholderia* resistance genes were exclusively found in the CF cohort, while *Haemophilus* resistance genes were solely identified in the NCFB cohort. The healthy cohort exhibited a more concise profile, where *mel, tet(Q), and CfxA2* led to resistance to only one drug class. There was a clear alignment between resistome data and microbiological resistance determined in patients (Table 2). The data compiled from the resistome analysis related to microbial resistance in patients be_05, cf_07, and cf_10 are displayed in Table 4.

**Table 3:** Most abundant resistance genes in the CF, NCFB and healthy cohorts

Cohort	Abundance (%)	ARO term	Pathogens with observed resistome variants	Drug class	Resistance mechanism
NCFB	10.29	hmrM	*Haemophilus influenzae*	Fluoroquinolone, disinfecting agents, antiseptics	Antibiotic efflux
	3.36	MexB	*Pseudomonas aeruginosa*	Macrolide, fluoroquinolone, carbapenem, tetracycline, peptide, beta-lactams	Antibiotic efflux
	3.17	MexK	*Pseudomonas aeruginosa*	Macrolide, tetracycline, disinfecting agents, antiseptics	Antibiotic efflux
	3.04	LpsA	*Haemophilus influenzae*	Peptide	Reduced permeability to antibiotics
	2.99	mexN	*Pseudomonas aeruginosa*	Phenicol	Antibiotic efflux
	2.87	mel	*Streptococcus pyogenes*	Macrolide, streptogramin	Antibiotic target protection
	2.78	MexF	*Pseudomonas aeruginosa*	Fluoroquinolone, diaminopyrimidine, phenicol	Antibiotic efflux
	2.74	MexI	*Pseudomonas aeruginosa*	Fluoroquinolone, tetracycline, disinfecting agents, antiseptics	Antibiotic efflux
	2.65	MexW	*Pseudomonas aeruginosa*	Macrolide, fluoroquinolone, tetracycline, phenicol, disinfecting agents, antiseptics	Antibiotic efflux
	2.65	MuxC	*Pseudomonas aeruginosa*	Macrolide, monobactam, tetracycline, aminocoumarin	Antibiotic efflux
CF	9.44	ceoB	*Burkholderia cepacia*	Fluoroquinolone, aminoglycoside	Antibiotic efflux
	6.35	amrB	*Burkholderia pseudomallei*	Aminoglycoside	Antibiotic efflux
	3.51	opcM	*Burkholderia cepacia*	Fluoroquinolone, aminoglycoside	Antibiotic efflux
	3.29	ceoA	*Burkholderia cepacia*	Fluoroquinolone, aminoglycoside	Antibiotic efflux
	3.23	MexB	*Pseudomonas aeruginosa*	Macrolide, fluoroquinolone, carbapenem, tetracycline, peptide, beta-lactams	Antibiotic efflux
	2.91	Omp38	*Burkholderia pseudomallei*	Cephalosporin, beta-lactams	Reduced permeability to antibiotics
	2.33	MexK	*Pseudomonas aeruginosa*	Macrolide, tetracycline, disinfecting agents, antiseptics	Antibiotic efflux
	2.28	MuxB	*Pseudomonas aeruginosa*	Macrolide, monobactam, tetracycline, aminocoumarin	Antibiotic efflux
	2.28	MexF	*Pseudomonas aeruginosa*	Fluoroquinolone, diaminopyrimidine, phenicol	Antibiotic efflux
	2.23	MuxC	*Pseudomonas aeruginosa*	Macrolide, monobactam, tetracycline, aminocoumarin	Antibiotic efflux
Healthy	11.74	mel	*Streptococcus pyogenes*	Macrolide, streptogramin	Antibiotic target protection
	11.38	tet(Q)	*Bacteroides fragilis*	Tetracycline	Antibiotic target protection
	9.87	rpoB mutation	*Bifidobacterium adolescentis*	Rifamycin	Antibiotic target alteration, antibiotic target replacement
	6.32	CfxA2	*Prevotella intermedia*	Cephamycin	Antibiotic inactivation
	5.15	tetA(46)	*Streptococcus australis*	Tetracycline	Antibiotic efflux
	5.06	lsaC	*Streptococcus agalactiae*	Lincosamide, streptogramin, pleuromutilin	Antibiotic target protection
	4.62	tetB(46)	*Streptococcus australis*	Tetracycline	Antibiotic efflux
	4	patA	*Streptococcus pneumoniae*	Fluoroquinolone	Antibiotic efflux
	3.95	tet(M)	*Erysipelothrix rhusiopathiae*	Tetracycline	Antibiotic target protection
	3.32	patB	*Streptococcus pneumoniae*	Fluoroquinolone	Antibiotic efflux

**Table 4:** Antimicrobial resistance genes identified.

**Subject**	**Pathogens with Resistome Variants**	**Drug class**	**% reads**
be_05	*Streptococcus pneumoniae*	Fluoroquinolone	3.658
	*Neisseria meningitidis*	Beta-lactam	1.219
cf_07	*Staphylococcus aureus*	Fluoroquinolone	54.067
cf_10	*Achromobacter insuavis*	Cephalosporin	4.608
	*Pseudomonas aeruginosa*	Cephalosporin	11.743

## Discussion

In the present study, CF and NCFB were selected since both diseases share clinical practices and characteristics. It is important to recognize that this similar clinical approach may not be universally effective for both conditions. The progression of CF and NCFB is closely tied to infectious processes. In this context, microbiome dysbiosis holds promise as a distinctive marker of overall patient progression [38]. The disease cohorts displayed a decreased alpha diversity compared to the healthy cohort, indicating a decline in species diversity within the pathological cohorts. Previous studies have already established a diminished diversity linked to lung pathological states [39]. This reduction is intricately tied to the frequency of replication of specific taxa, which disrupts the balance between migration and replication dynamics associated with the impact of antimicrobial therapy [40]. Despite the observed differences between the healthy and disease cohorts, CF and NCFB subjects exhibited similar beta diversities. Thus, regardless of their origins, both disease states presented a comparable number of observed distinctions.

When assessing the microbial communities in the lower respiratory tract, we found significant differences between the disease and healthy cohorts. CF and NCFB patients are known to exhibit a similar microbiological composition [2]; thus, the comparable number of observed distinctions in our study due to the lack of beta diversity among the disease cohorts aligns with previous reports. Moreover, a significantly elevated intra-cohort Bray–Curtis dissimilarity was found between the CF and NCFB cohorts, underscoring the heterogeneous microbiome composition in these individuals, also previously documented [41].

The nasopharyngeal microbiomes were consistently similar among the three cohorts. Within the context of the disease’s pathophysiology, in which changes in mucus rheology and lung morphology play a pivotal role in determining infection susceptibility [2, 4], the nasopharynx function remains relatively less affected. Although our findings showed no differences in the microbiome of the upper respiratory tract between the disease and healthy cohorts, this site is known to be an important pathogen reservoir [42]. The lack of differences in nasopharyngeal swab samples between our studied cohorts may be due to the superficial nature of this type of collection. Another study showed that nasal lavage samples correlated with clinical data, while nasal swab samples did not show the same relationship in CF patients [43]. Considering the impact of highly effective modulator therapies in patients with CF, which substantially affects sputum production, it is fundamental to find alternative samples for the clinical follow-up of these patients. In our study, the nasopharyngeal swabs did not differentiate the cohorts in terms of microbiome composition and diversity, highlighting the need for more studies to evaluate other types of samples for patients with reduced expectoration such as oropharyngeal or cough swabs, sinus aspirates, and other upper respiratory tract samples in these cohorts.

The studied healthy cohort exhibited higher counts of Actinobacteria, Fusobacteria, Firmicutes, and Bacteroidetes. Phylum Actinobacteria is enriched in healthy individuals and in mild cases of asthma compared to severe cases [44]. In healthy subjects, enrichment of the Firmicutes phylum in young adults displayed a positive correlation with lung function [45]. Fusobacteria have also been described as positively correlated with lung spirometry parameters in various conditions [46]. Nevertheless, our findings did not reveal a correlation between the frequency of these phyla and lung function in CF and NCFB, possibly due to the limited number of subjects in each cohort. Both compositional analysis and phylum-level comparisons revealed a trend toward Proteobacteria enrichment in the disease cohorts, particularly in CF, compared to the healthy cohort. This association aligns with the hypothesis that Proteobacteria are intimately linked to the disease state and inflammation [47].

The core microbiota components reinforced the distinction between disease cohorts and healthy subjects. In the healthy cohort, the core components consisted of well-established genera in the lungs and upper respiratory tract [48]. All components of the core CF cohort were also present in the NCFB cohort, reinforcing the similarity between CF and NCFB microbiota.

Members of the *Burkholderiaceae* family, particularly the *Burkholderia cepacia* complex (BCC), were also enriched in the CF cohort compared to the healthy cohort. This finding is in agreement with clinical observations, as the BCC is closely associated with CF and known to correlate with lung failure [49]. The *Rhizobium* genus, typically linked to nitrogen fixation in plants, has been described in CF patients without apparent effects on lung functionality [50]. Our findings suggest its high prevalence in both CF and NCFB. *Cutibacterium* (formerly *Propionibacterium*) is also found in the upper respiratory tract [51], but in this study, it emerged as a major component of the lung microbiota of both disease cohorts. The NCFB cohort also exhibited three exclusive genera in its core: *Capnocytophaga*, *Lautropia*, and *Treponema*. The presence of *Capnocytophaga* and the reduction of *Treponema* are associated with COPD exacerbation [52, 53], while *Lautropia* is linked to lung dysbiosis in CF and arterial stiffness in young adults [45].

The differential abundance analysis unveiled a high number of differential species when comparing the healthy and CF cohorts, underscoring the pronounced distinctions between them. The microorganisms enriched in the healthy cohort were associated with the upper respiratory tract [48, 54]. This observation further fortifies the notion that migration plays a predominant role in shaping the lung microbiome in healthy subjects [10, 39]. The presence of *Ackermannviridae* sp. phage is of notable significance. Phages have attracted attention in recent years as they have been identified as constituents of the human microbiome and the most prevalent viruses in the lungs [55]. *Ackermannviridae* sp. has primarily been characterized within the gut microbiota, with its role in the lungs being less understood [56]. Phages are hypothesized to potentially act to eliminate pathogens, functioning in a defensive capacity [57]. Therefore, *Ackermannviridae* sp. presence may potentially correlate positively with protection against infection.

In the CF cohort, differentially abundant bacteria majorly included members of the *Burkholderiaceae* family, including *Ralstonia* sp. associated with *Pseudomonas* and an unclassified *Xanthomonas* [58]. As *Burkholderiaceae* and *Pseudomonas* are part of phylum Proteobacteria, these findings correlate with the enrichment of Proteobacteria in CF subjects compared to the healthy cohort. The presence of the genera *Pseudomonas* and *Ralstonia* in CF subjects is a common observation [15, 59], as *P. aeruginosa* is often associated with the dominance of the microbiome in CF cases and linked to unfavorable outcomes [11]. Chronic *P. aeruginosa* infection is also challenging to treat considering biofilm formation and the microorganism’s capability to adapt to stressful conditions [60]. *Ralstonia*, on the other hand, consists of opportunistic pathogens, typically displaying intrinsic resistance patterns to antimicrobial agents [61].

The BCC was also identified as being more abundant in CF patients than in NCFB patients. The increased susceptibility to the BCC in CF subjects may be linked to factors extending beyond lung alterations. The syndromic aspects of CF can impact the autophagy process, and BCC components have been shown to evade elimination by subverting autophagy mechanisms [62]. *CFTR* mutations lead to an increased susceptibility to this mechanism, resulting in infection perpetuation in CF patients, whereas individuals with NCFB eliminate BCC infection more efficiently [62].

When examining the differential abundance of species in NCFB subjects compared to the healthy cohort, *P. aeruginosa* prominently emerged. This result is in agreement with clinical observations since *P. aeruginosa* is also a prevalent pathogen in NCFB, closely associated with disease severity, similar to its role in CF [12, 63]. In contrast, when we compared NCFB and CF subjects, *H. influenzae,* a classical bronchiectasis pathogen [12], and *P. shahii* exhibited increased abundance in the NCFB cohort. Since *Prevotella* is a constituent of the oral commensal microbiota [64], this prompts questions about whether NCFB exhibits a more pronounced influence of migration on microbiota composition than CF.

Despite the differential abundance of various species between cohorts, functional analysis revealed distinctions only between the CF and healthy cohorts. Consistent with previous findings, in this study, the healthy cohort displayed enrichment of Bacteroidetes proteins [65, 66], reinforcing its higher composition when compared to CF. In the CF cohort, we observed the enrichment of proteins related to basic metabolism, indicating heightened metabolic activity. Additionally, we found an association with proteins linked to virulence and pathogenicity, such as MCP (related to quorum sensing [67]), antimicrobial resistance factors like LysR transcription regulators [68] and MarR [69], as well as stress response proteins [70]. Collectively, these findings demonstrate the adaptation of the CF cohort lung microbiota to the hostile environment compared to the healthy cohort, suggesting a colonization characteristic in CF microbiota.

To assess antimicrobial resistance and possible correlations with potential clinical implications, we examined the composition and frequency of resistance genes in the three cohorts. Both disease cohorts displayed multidrug resistance genes, associated with highly prevalent pathogens, such as *MexB* and *MexW*, both encoding efflux pumps in *P. aeruginosa* [71, 72]. Together, these findings indicate a high prevalence of resistance associated with the most common pathogen in both CF and NCFB [73, 74].

In sputum samples, healthy and NCFB subjects displayed a lower frequency of resistance genes when compared to the CF cohort. Given that both diseases involve comparable clinical approaches for antimicrobial administration [75], this finding, together with the compositional differences between the two disease cohorts, was intriguing. This difference may potentially be linked to variations in the prevalence of pathogens in both disease cohorts. While *P. aeruginosa* displayed similar resistance genes in both diseases, the CF cohort exhibited an enriched composition of resistance genes related to the *Burkholderia* genus. Members of the *Burkholderia* genus also showed a log_2_FC in the range of 20-25. Collectively, this may explain the higher incidence of resistance genes in the CF compared to the NCFB and healthy cohorts.

The potential application of metagenomics data for tailoring treatments to individual patients has been previously emphasized [76]. Our data further substantiate this potential, showing a clear alignment between the identified resistome data and the clinical data of patients be_05, cf_07, and cf_10. The majority of genes related to drug resistance in those three patients encoded drug efflux pumps. This finding aligns with the observation of an enriched efflux resistance mechanism. It is important to emphasize the significance of efflux pumps, especially in these patients, given that efflux pumps alone can lead to multidrug resistance [77].

Using a comprehensive approach and metagenomic next-generation sequencing, we assessed the microbial composition and frequency of antimicrobial resistance genes in CF, NCFB, and healthy cohorts, considering the prolonged antibiotic exposure experienced by patients with these diseases. A limitation of our study is the cross-sectional design, which did not allow for patient’s follow-up, as well as the limited access to their clinical data. Regarding the metagenomic analysis, besides the small sample size for each cohort and the absence of a metagenomic mock sample, our results highlight the disparities of microbiome structures and potential clinical implications for each disease. Despite the low percentage of sequenced microbial DNA, all samples reached a plateau of identified taxa and resistance gene markers according to the rarefaction curves. However, this low proportion may hinder the complete determination of each patient’s resistome. Our findings have significant clinical relevance and reinforces the importance of personalized treatment strategies for patients with CF and NCFB.

## Conclusions

**Fig. 7:**
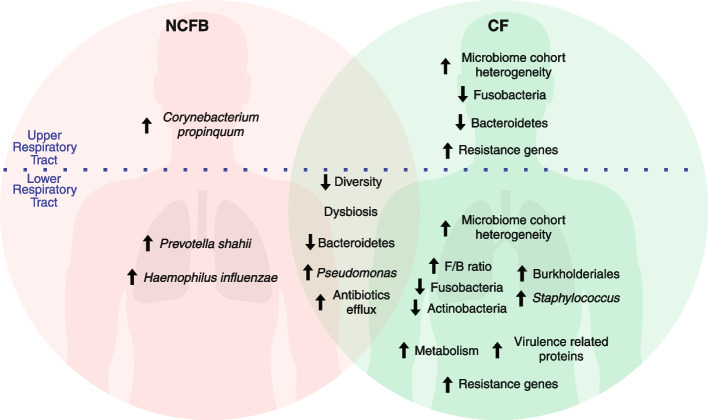
Impact of NCFB and CF on the respiratory tract microbiome. In the upper respiratory tract, NCFB is associated with an increase in *C. propinquum*. The CF cohort presents heightened microbiome cohort heterogeneity, decreased Fusobacteria and Bacteroidetes levels, and increased presence of resistance genes. In the lower respiratory tract, both NCFB and CF result in reduced diversity, lower Bacteroidetes phylum levels, an increase in the *Pseudomonas* genus, and elevated levels of antibiotic efflux mechanisms among resistance genes. The NCFB cohort exhibits an increased prevalence of *P. shahii* and *H. influenzae* compared to the CF cohort. The CF cohort is characterized by increased microbiome cohort heterogeneity, a rise in the Burkholderiales order, augmented *Staphylococcus* genus levels, an elevated Firmicutes/Bacteroidetes ratio, and diminished Fusobacteria and Actinobacteria. CF also manifests increased markers of metabolic activity, heightened levels of virulence-related proteins, and an increased presence of resistance genes

Our study revealed distinct microbial compositions and resistance gene profiles in CF and NCFB patients compared to healthy subjects. While some similarities existed between the disease cohorts, CF had a more pronounced impact on the lung microbiome, evidenced by greater dissimilarity to the healthy cohort. Both the CF and NCFB groups showed reduced species diversity, likely influenced by antimicrobial therapy and the dominance of specific pathogens. Additionally, the CF and healthy cohorts displayed significant differences in the abundance of functional processes. The nasopharynx exhibited a consistent microbiota composition across all cohorts, suggesting limited diagnostic value for this site. Although CF and NCFB shared some similarities, notable differences existed in microbial composition, with the *Burkholderiaceae* family playing a crucial role in distinguishing them. Antimicrobial resistance gene profiles also significantly differed between cohorts. Our data establish a connection between resistome data and clinical observations, highlighting the potential for molecular approaches to guide therapy. Our findings are summarized in Fig. 7 and underscore the importance of tailored strategies for each disease, primarily antimicrobial agent selection in the context of antimicrobial resistance.

### Supplementary Information

Supplementary Material 1: Figure S1. Rarefaction curves of identified operational taxonomic units (OTUS) and resistance genes. A and B. Identified OTUS in sputum (A) and nasopharyngeal (B) samples. C and D. Identified resistance genes in sputum (C) and nasopharyngeal (D) samples. The X-axis represents the sample reads. Curves in pink denote NCFB, green denotes CF, and blue denotes healthy patients.

Supplementary Material 2: Figure S2. Alpha diversity comparison between NCFB, CF, and healthy cohorts. A and B. Simpson index and evenness for sputum (A) and nasopharyngeal samples (B). Statistical significance: Kruskal–Wallis test followed by Dunn’s post hoc test (** *P* ≤ 0.01).

Supplementary Material 3: Figure S3. Linear regression of CF sputum phyla counts and FEV1. The correlation between phyla and lung function was assessed through linear regression with phylum counts. The linear regression line is represented in red, and its standard error is shown as a darker area on the graph.

Supplementary Material 4: Figure S4. Linear regression of NCFB sputum phyla counts and FEV1. The correlation between phyla and lung function was assessed through linear regression with phylum counts. The linear regression line is represented in red, and its standard error is shown as a darker area on the graph.

Supplementary Material 5: Figure S5. Pairwise differential species abundance analysis volcano plots. A-C. Differential species abundance analysis comparing sputum samples of healthy subjects and individuals with CF (A), healthy subjects and individuals with NCFB (B), and CF and NCFB subjects (C). D-F. Differential abundance analysis comparing nasopharynx samples of healthy subjects and individuals with CF (D), healthy subjects and individuals with NCFB (E), and CF and NCFB subjects (F). DESeq2 was employed for the analysis, with significance defined by an adjusted *p* value below 0.01. The figure represents differentially abundant species with a log_2_FC of 5 for the healthy *vs.* CF comparison and a log_2_FC of 4 for the other comparisons.

Supplementary Material 6. Table_S1 to Table_S11.

## Data Availability

Metagenomic data files are available at NCBI GenBank under accession number PRJNA1055940.
