# Supplementary figures and images for: Comparative microbiome analysis in cystic fibrosis and non-cystic fibrosis bronchiectasis
Source: Respir Res. 2024 May 18;25:211. doi: 10.1186/s12931-024-02835-w (PMC11102160; doi:10.1186/s12931-024-02835-w)

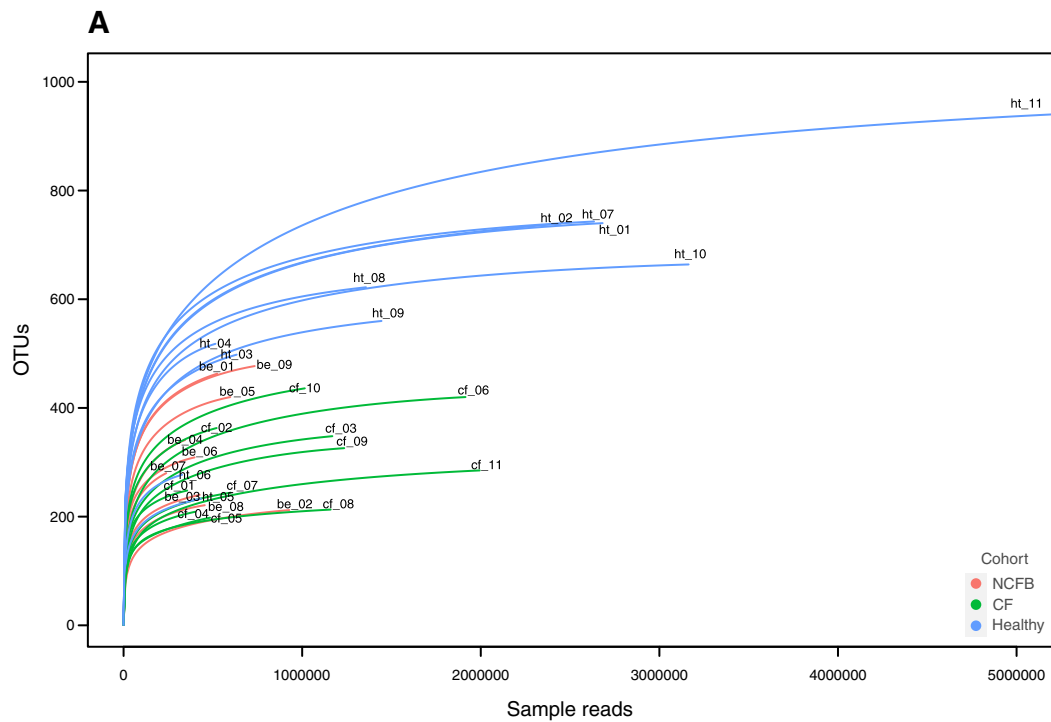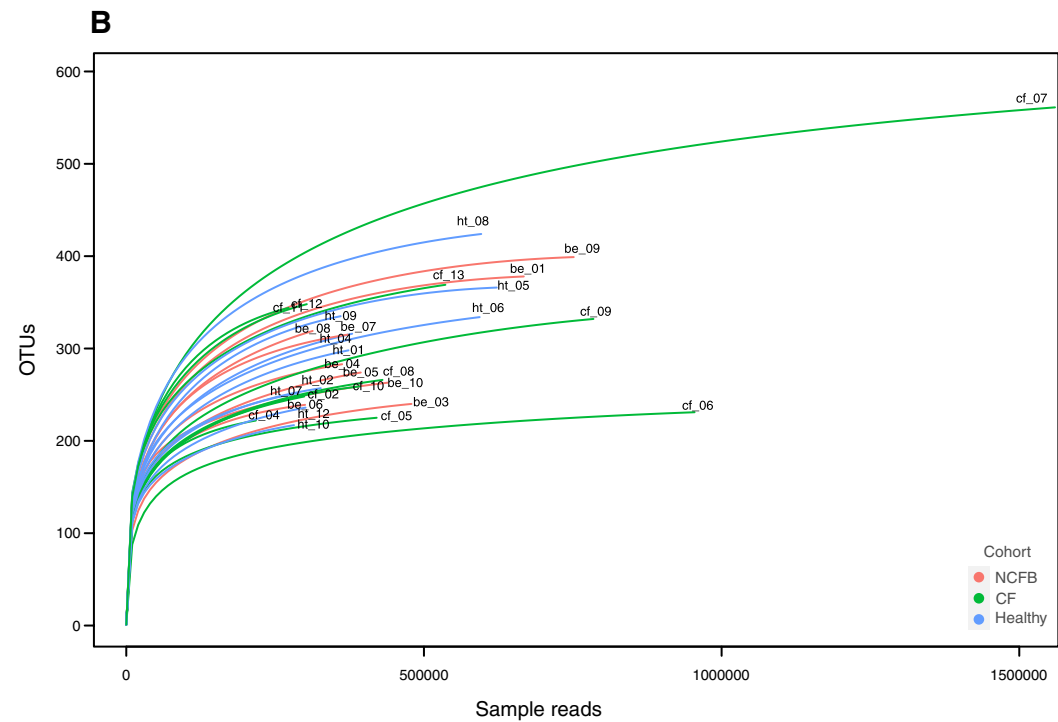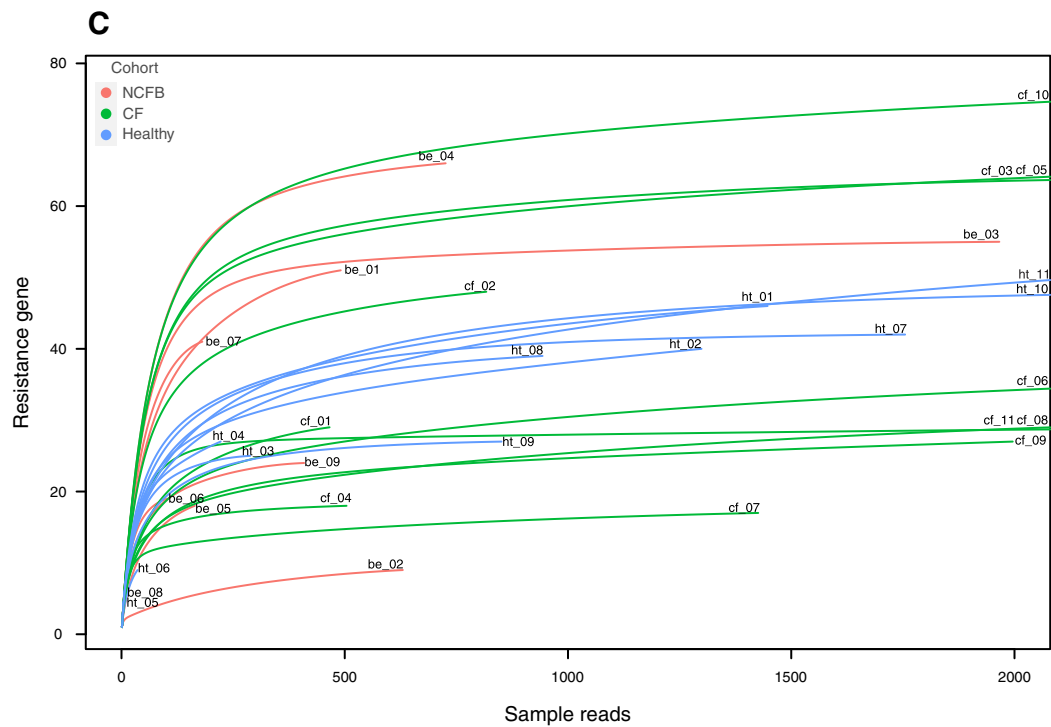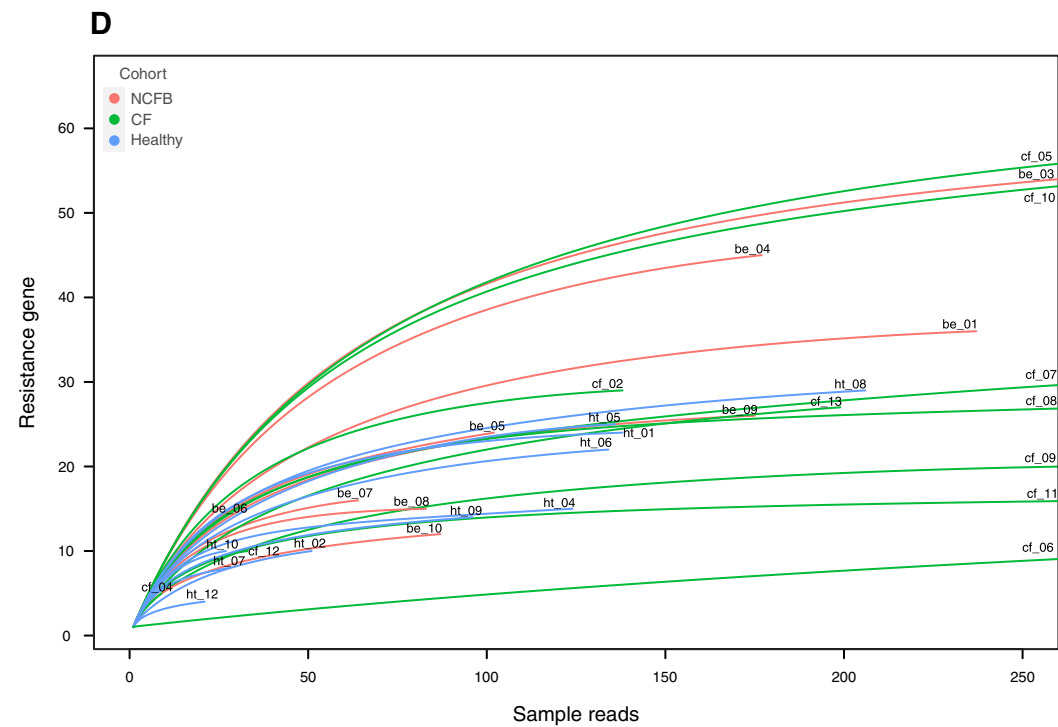

Supplement: Supplementary file 1 — Supplementary Material 1: Figure S1. Rarefaction curves of identified operational taxonomic units (OTUS) and resistance genes. A and B. Identified OTUS in sputum (A) and nasopharyngeal (B) samples. C and D. Identified resistance genes in sputum (C) and nasopharyngeal (D) samples. The X-axis represents the sample reads. Curves in pink denote NCFB, green denotes CF, and blue denotes healthy patients. [file 12931_2024_2835_MOESM1_ESM.pdf]

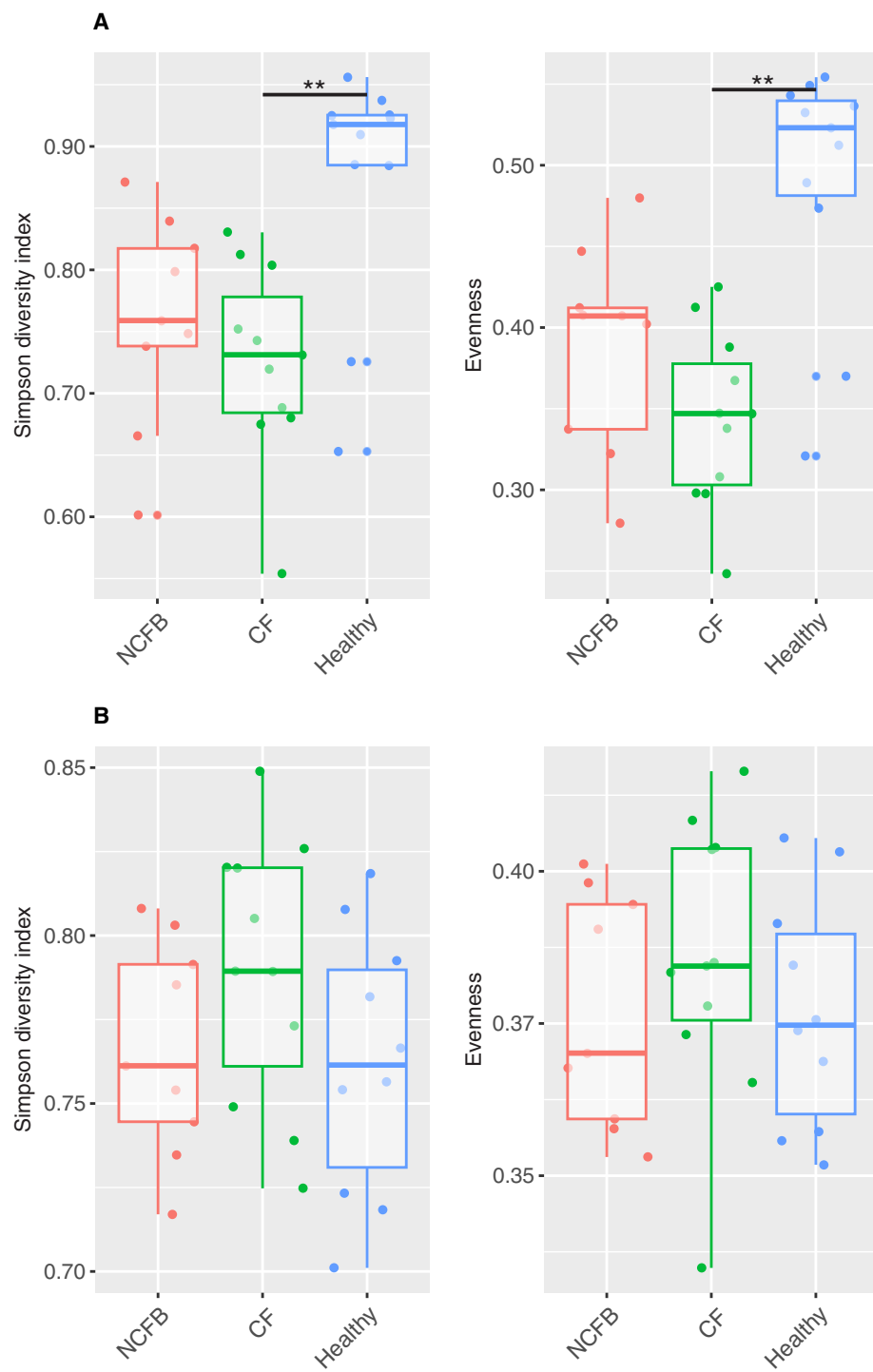

Supplement: Supplementary file 2 — Supplementary Material 2: Figure S2. Alpha diversity comparison between NCFB, CF, and healthy cohorts. A and B. Simpson index and evenness for sputum (A) and nasopharyngeal samples (B). Statistical significance: Kruskal–Wallis test followed by Dunn’s post hoc test (** P ≤ 0.01). [file 12931_2024_2835_MOESM2_ESM.pdf]

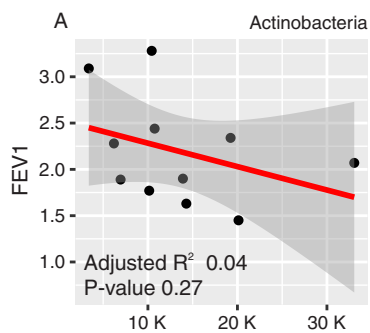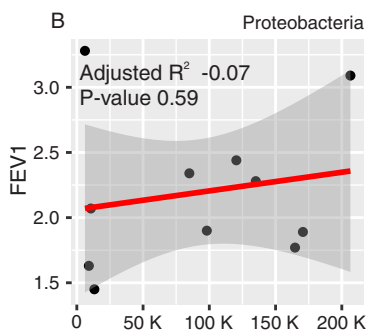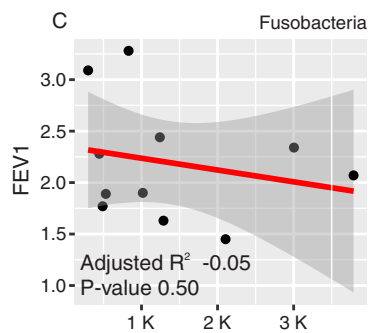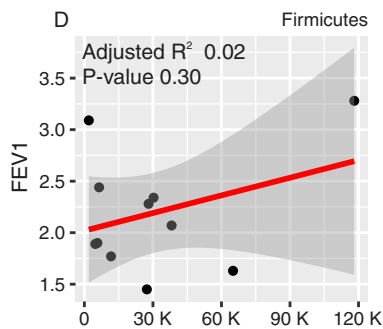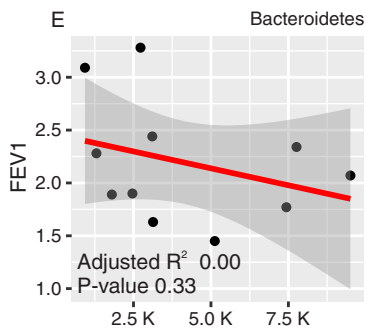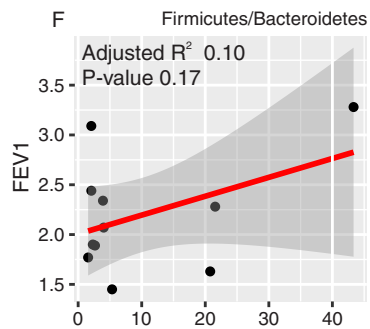

Supplement: Supplementary file 3 — Supplementary Material 3: Figure S3. Linear regression of CF sputum phyla counts and FEV1. The correlation between phyla and lung function was assessed through linear regression with phylum counts. The linear regression line is represented in red, and its standard error is shown as a darker area on the graph. [file 12931_2024_2835_MOESM3_ESM.pdf]

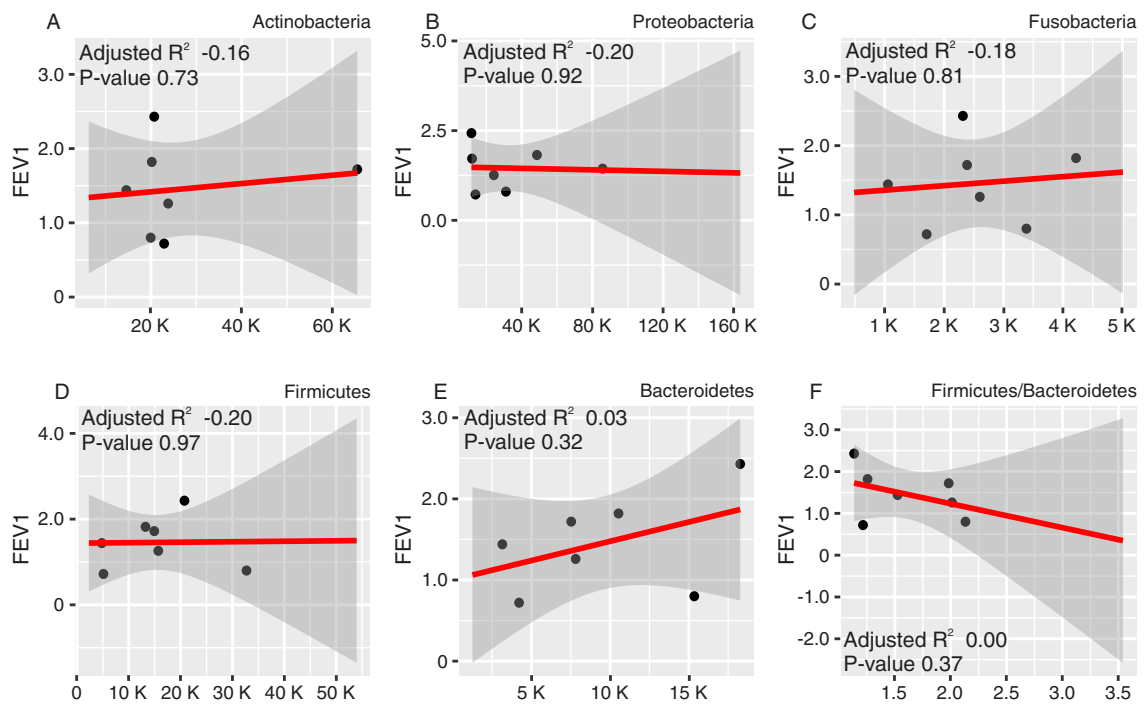

Supplement: Supplementary file 4 — Supplementary Material 4: Figure S4. Linear regression of NCFB sputum phyla counts and FEV1. The correlation between phyla and lung function was assessed through linear regression with phylum counts. The linear regression line is represented in red, and its standard error is shown as a darker area on the graph. [file 12931_2024_2835_MOESM4_ESM.pdf]

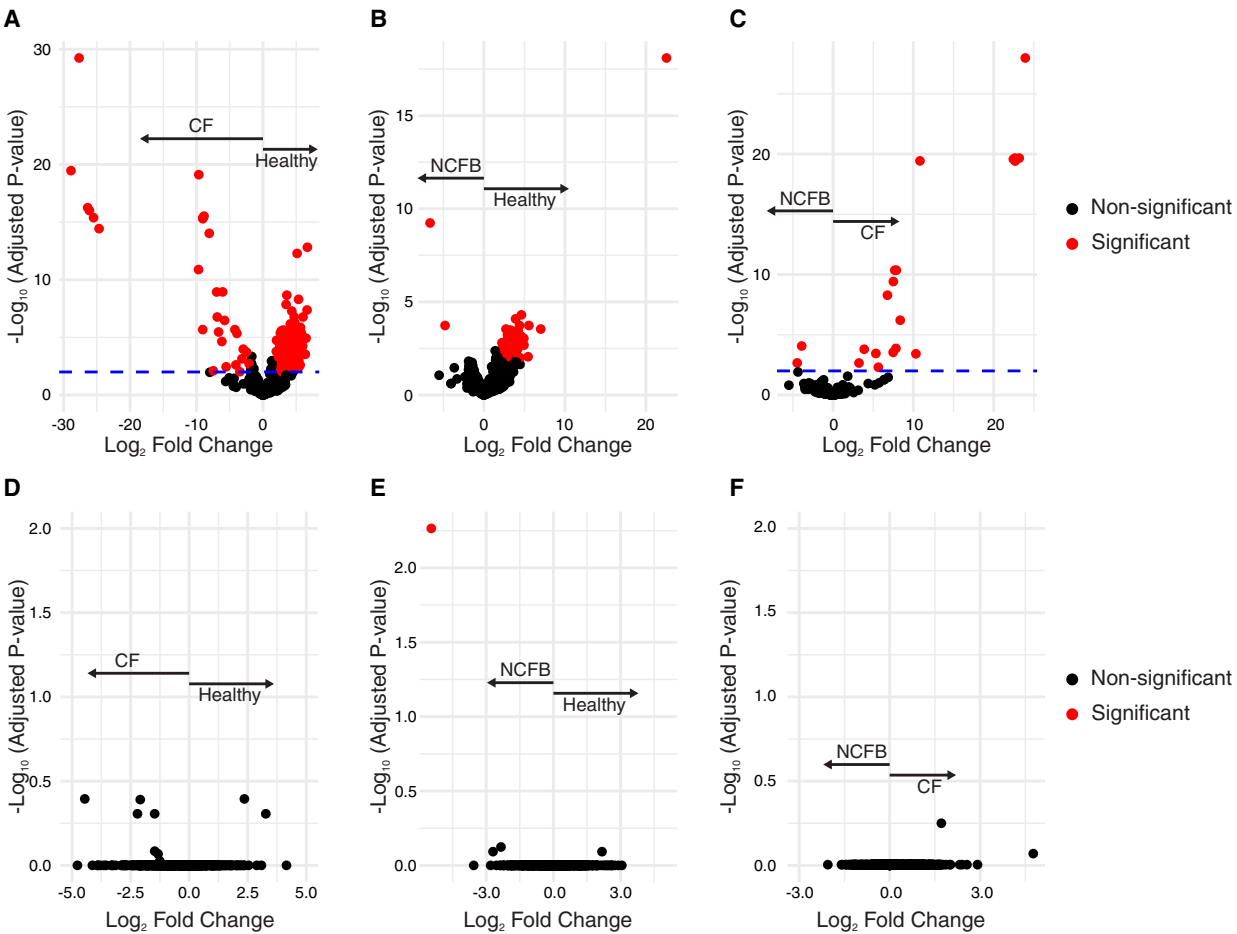

Supplement: Supplementary file 5 — Supplementary Material 5: Figure S5. Pairwise differential species abundance analysis volcano plots. A-C. Differential species abundance analysis comparing sputum samples of healthy subjects and individuals with CF (A), healthy subjects and individuals with NCFB (B), and CF and NCFB subjects (C). D-F. Differential abundance analysis comparing nasopharynx samples of healthy subjects and individuals with CF (D), healthy subjects and individuals with NCFB (E), and CF and NCFB subjects (F). DESeq2 was employed for the analysis, with significance defined by an adjusted p value below 0.01. The figure represents differentially abundant species with a log2FC of 5 for the healthy vs. CF comparison and a log2FC of 4 for the other comparisons. [file 12931_2024_2835_MOESM5_ESM.pdf]
